# Scalp incision technique for decompressive hemicraniectomy: comparative systematic review and meta-analysis of the reverse question mark versus alternative retroauricular and Kempe incision techniques

**DOI:** 10.1007/s10143-024-02307-1

**Published:** 2024-02-14

**Authors:** Nolan J. Brown, Julian Gendreau, Redi Rahmani, Joshua S. Catapano, Michael T. Lawton

**Affiliations:** 1 Department of Neurological Surgery, University of California-Irvine, Orange, CA USA; 2 Department of Biomedical Engineering, Johns Hopkins Whiting School of Engineering, Baltimore, MD USA; 3 Department of Neurosurgery, Barrow Neurological Institute, St. Joseph’s Hospital and Medical Center, 2910 North Third Avenue, Phoenix, AZ 85013 USA

**Keywords:** Neurosurgery, Cranial trauma, Decompressive hemicraniectomy, TBI, Refractory ICP, Herniation, Temporal fossa decompression, Scalp incision, Bone flap, Reverse question mark, Wound complications, Surgical site infection

## Abstract

Decompressive hemicraniectomy (DHC) is a critical procedure used to alleviate elevated intracranial pressure (ICP) in emergent situations. It is typically performed to create space for the swelling brain and to prevent dangerous and potentially fatal increases in ICP. DHC is indicated for pathologies ranging from MCA stroke to traumatic subarachnoid hemorrhage—essentially any cause of refractory brain swelling and elevated ICPs. Scalp incisions for opening and closing the soft tissues during DHC are crucial to achieve optimal outcomes by promoting proper wound healing and minimizing surgical site infections (SSIs). Though the reverse question mark (RQM) scalp incision has gained significant traction within neurosurgical practice, alternatives—including the retroauricular (RA) and Kempe incisions—have been proposed. As choice of technique can impact postoperative outcomes and complications, we sought to compare outcomes associated with different scalp incision techniques used during DHC. We queried three databases according to PRISMA guidelines in order to identify studies comparing outcomes between the RQM versus “alternative” scalp incision techniques for DHC. Our primary outcome of interest in the present study was postoperative wound infection rates according to scalp incision type. Secondary outcomes included estimated blood loss (EBL) and operative duration. We identified seven studies eligible for inclusion in the formal meta-analysis. The traditional RQM technique shortened operative times by 36.56 min, on average. Additionally, mean EBL was significantly lower when the RQM scalp incision was used. Postoperatively, there was no significant association between DHC incision type and mean intensive care unit (ICU) length of stay (LOS), nor was there a significant difference in predisposition to developing wound complications or infections between the RQM and retroauricular/Kempe incision cohorts. Superficial temporal artery (STA) preservation and reoperation rates were collected but could not be analyzed due to insufficient number of studies reporting these outcomes. Our meta-analysis suggests that there is no significant difference between scalp incision techniques as they relate to surgical site infection and wound complications. At present, it appears that outcomes following DHC can be improved by ensuring that the bone flap is large enough to enable sufficient cerebral expansion and decompression of the temporal lobe, the latter of which is of particular importance. Although previous studies have suggested that there are several advantages to performing alternative scalp incision techniques during DHC, the present study (which is to our knowledge the first to meta-analyze the literature on outcomes in DHC by scalp incision type) does not support these findings. As such, further investigations in the form of prospective trials with high statistical power are merited.

## Introduction

Decompressive hemicraniectomy (DHC) is an emergent procedure used to provide room for the swelling brain to expand while avoiding potentially fatal increases in ICP [1, 2]. Indications include ischemic infarct resulting from stroke of the middle cerebral artery (MCA) or malignant MCA occlusion syndrome. Additionally, DHC can be performed in the setting of uncontrollable brain swelling after craniotomy has already been performed [1–3]. Furthermore, any cause of traumatic intracranial hypertension, including epidural (EDH), traumatic subarachnoid hemorrhage (tSAH), and/or subdural hematomas (SDH), often require emergency surgery in which decompression is warranted. [4–6]

Even though this invasive procedure is not without its complications, it is seen as a lifesaving treatment. However, it is only performed when the benefits outweigh potential risks [7]. Such risks include postoperative bleeding, herniation of the brain matter though the calvarial opening if it is not made large enough, risk for injury to the decompressed and exposed brain (with the bone flap removed), and any postoperative fluid collections such as hematomas at the operative site and subdural hygromas.

In addition to its standard uses for severe TBI and malignant cerebral ischemia, the procedure is becoming more commonplace as a means by which to address aneurysmal subarachnoid hemorrhage (aSAH) [8]. Currently, it is also undergoing investigation as a treatment modality for severe intracerebral hemorrhage (ICH) [9]. Regardless of presenting etiology of elevated ICP, cranioplasty is required to repair the defect of the calvarium once brain swelling has subsided. [10]

In summation, DHC is one of the most commonly performed procedures intended to relieve refractory elevated ICPs, often in the setting of intracranial hemorrhage, within neurological surgery [11]. Although common, it is a last resort treatment for these cases of refractory intracranial hypertension as it has a high rate of perioperative morbidity; unfortunately, a large portion of adverse outcomes result from wound healing disturbances as opposed to the general success of the surgery itself [8]. Scalp incisions for opening and closure of the soft tissues required to expose the cranial vault are thus arguably among the most important steps of the procedure, as the manner in which they are performed has a large effect on postoperative healing and wound infection rates [8]. It is also paramount that the craniectomy be large enough to accommodate further brain expansion to avoid brain herniation along the edges of a craniectomy that is too small [8]. Given the importance of careful, precise incision and closure to ensure optimal closure and healing of the scalp incision, it is relevant to consider the different scalp incision techniques that have been proposed over the years. In the present study, we compare outcomes and complications associated with emergent decompression in which the reverse question mark (RQM) incision is used as compared to alternative techniques, such as the retroauricular and Kempe incisions. In doing so, we hope to shed light on the benefits and drawbacks of each technique and potentially draw attention to any indications that merit use of an alternative scalp incision technique over the traditional RQM incision and “Dandy” flap.

## Methods

### Search strategy

To identify all studies comparing outcomes following decompressive hemicraniectomy using the reverse question mark versus alternative scalp incision techniques, a literature search was conducted according to PRISMA guidelines using the PubMed, Scopus, and Web of Science databases. Specifically, we queried each database using the following Boolean search term: (decompressive) AND (craniectomy OR hemicraniectomy) AND (scalp incision OR reverse question mark OR question mark OR retroauricular OR Dandy’s OR Kempe OR T-shaped).

### Selection criteria

After all three database were queried, duplicate studies were removed and all non-duplicate records returned by the search were screened according to title and abstract using the following inclusion criteria: (1) primary study featuring a clinical series or retrospective cohort, (2) comparing the reverse question mark scalp incision to other techniques, including the retroauricular and Kempe (T-type) incision or retroauricular incision, (3) for the performance of decompressive hemicraniectomy by cranial neurosurgeons. All studies meeting any of the following criteria were excluded from the present analysis: (1) studies not written in English language or a suitable English translation, (2) those not available in full-text form, (3) non-comparative studies, (4) lack of inclusion of the reverse question mark scalp incision as the control technique for comparison, and (5) systematic reviews, editorials, letters to the editor, and any non-primary forms of clinical research studies.

### Study appraisal and quality assessment

Following establishment of these criteria, search results were screened against title and abstract by two reviewers. If the reviewers disagreed on the inclusion/exclusion of a given study, resolution was pursued through the consultation of a third author who served as arbitrator until consensus decision was reached. Upon completion of the title and abstract screen, full texts for each study were scrutinized to determine the potential suitability of each study for inclusion in the final review. Furthermore, the references of all included studies were examined to identify any additional studies bearing relevance to our analysis that may have been missed during initial screening steps.

Next, every study meeting criteria for inclusion in the present study was assigned a risk of bias rating using the Newcastle–Ottawa Scale (NOS) scoring guidelines. The NOS criteria allow for a maximum of four points in *selection*, two points in *comparability*, and three points in *outcome:* the total range was 0–9. The results of this assessment along with a Level of Evidence designation for each study are listed in Table 1. A Levels of Evidence categorization system developed by the Oxford Center for Evidence-Based Medicine was utilized; this system assigns quality scores on a scale ranging from I (highest) to V (lowest). Level I studies consist of randomized, controlled trials (RCTs) of the highest caliber. Level II studies include RCTs with systematic or methodologic limitations, while Level III is assigned to most quality retrospective cohort studies. Most case series meet the criteria for Level IV evidence, while case reports and expert opinions are assigned Level V, the lowest quality evidentiary category.

**Table 1:** Patient demographics and characteristics of included studies

Study (author, year)	Cohort size (%male)	Mean age	Pathologies meriting decompression	Scalp incision techniques compared	Findings and conclusions	Level of Evidence/NOS ROB Score
Abecassis et al., 2021	163 (22.8%)	Craniectomy: RQM: 49.7 ± 15.9 Kempe: 52.8 ± 16.2	Traumatic aSDH: 17 Mixed TBI: 25 Mixed SDH: 1 Ischemic stroke: 15 Hemorrhagic stroke: 25	Craniotomy group: RQM (44) and Kempe (13) Craniectomy Group: RQM (41) and Kempe (38)	Kempe incision is safe and effective for both craniotomy and decompressive hemicraniectomy. Kempe may provide larger decompression than RQM	Evidence level: III, ROB: 6
Dowlati et al., 2021	106 (60.4%)	RQM (*n* = 63): 51.7 ± 14.6 RA (*n* = 43) 48.5 ± 15.0	CVA: 18 (28.6%) SAH: 1 (1.6%) Trauma: 6 (9.6%) IPH: 18 (28.6%) SDH: 17 (27.0%) Other: 1 (1.6%)	Classic RQM versus RA	The RA incision can increase calvarial exposure and is an attractive alternative to the classic RQM. It is safe and effective. It appears to enable decreased wound complications	Evidence level: III, ROB: 4
Eltabl et al., 2022	180 (total) RQM (80) RA (50) Kempe (50)	RQM: 45 ± 10.5 RA: 42 ± 12.2 Kempe: 50 ± 8.3	MCA infarction Infarction, multiple territories ICH IVH Cerebellar hemorrhage	Classic RQM versus RA versus Kempe	RA incision is safe substitute for the RQM and Kempe incisions in regard to maintaining blood flow and lower rate of post-operative wound complications	Evidence level: III, ROB: 6
Fruh et al., 2023	69 (40.6%)	51 ± 12	DHC for malignant hemispheric stroke (62.3%), ICH, and/or severe TBI (23.2%)	Classic RQM versus RA	RA provided better decompression of the temporal base and enabled preservation of the STA in all cases	Evidence level: III, ROB: 8
Nertengian et al., 2022	60	56.5	MCA infarction: 36 (60%) TBI: 11 (18.3%) Spontaneous ICH: 7 (11.7%) Aneurysmal SAH: 4 (6.7%) Venous sinus thrombosis: 2 (3.3%)	RQM “Dandy flap”: 30 RMF incision: 30	Shorter operative duration when RMF incision used	Evidence level: III, ROB:4
Veldemen et al., 2020	186	49 ± 16	Stroke (83 patients, 44.6%), traumatic brain injury (55 patients, 29.6%), subarachnoid hemorrhage (33 patients, 17.7%), and intracerebral hemorrhage (15 patients, 8.1%)	Classic RQM versus altered posterior question mark (RA)	The experimental retroauricular incision (posterior question-mark) provided benefits of reduced SSI and post-CP related complication. This benefit attributed to the superior vascularization of the skin afforded by a preserved STA and occipital artery, which was facilitated by the retroauricular incision technique	Evidence level: III, ROB: 7
Ordonez-Rubiano et al., 2021	10 (60%)	42.1 (range: 26–71)	TBI: 3 ICH: 2 Ischemic stroke: 2 Subdural empyema: 1	Classic RQM versus Kempe	Kempe appears to be equally effective to RQM. No differences noted between RQM and Kempe for scalp necrosis, infection, or cosmetic outcomes. Decision should be made on case-by-case basis	Evidence level: III, ROB: 3

### Data collection and statistical analysis

For all studies meeting the inclusion and exclusion criteria as outlined above, a standardized form was utilized to extract and store data from each study in an organized manner. Among the data collected were publication year, institutional affiliation and country of authors, study design, patient sample size, demographic factors (age and sex), specific reason and/or pathology meriting urgent decompressive craniectomy, specific scalp incision technique used, and any wound complications arising in the postoperative period (namely surgical site infection (SSI) and wound breakdown/dehiscence). Our primary outcome of interest in the present study was postoperative wound infection rates/SSI according to scalp incision type. Secondary outcomes included estimated blood loss (EBL) and operative duration.

Quantitative meta-analysis was performed according to the Mantel–Haenszel method using Review Manager v5.4 (Nordic Cochrane Centre, Cochrane Collaboration, Copenhagen, Denmark). Odds ratios (ORs) and mean differences (MDs) along with pooled 95% confidence intervals (CIs) were calculated to assess for the effect size of scalp incision technique on primary outcomes. Results were presented as forest plots, representing OR, MD, relative weights, and 95% CIs. Heterogeneity across studies was evaluated using the chi-square, *I*^2^, and *τ*^2^ tests. When *I*^2^ ≥ 50%, indicating substantial heterogeneity, a random effects model was used. Alternatively, when *I*^2^ < 50%, indicating relatively less heterogeneity across studies, a fixed effects model was used. Review Manager provided funnel plots specific to each outcome as a representation of the risk of bias and the relationship between cohort size and effect size. Throughout the analyses included in this study, all *p*-values < 0.05 were considered statistically significant.

## Results

### Study selection and levels of evidence of included studies

**Fig. 1:**
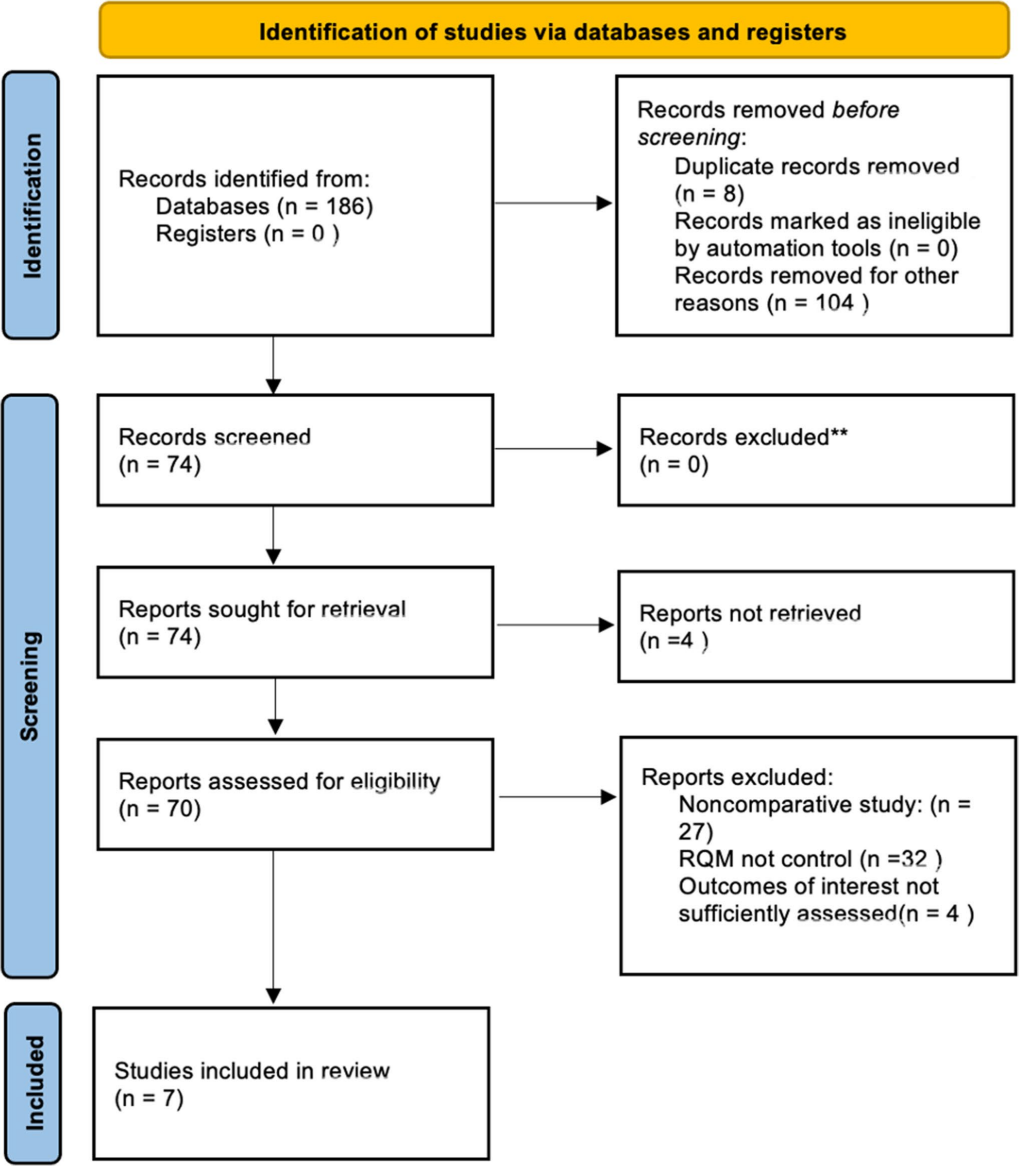
PRISMA flow diagram detailing study selection process

Our preliminary query yielded 74 results following the removal of duplicate studies. Following title and abstract screen as well as final application of inclusion/exclusion criteria during full-text review, seven studies remained eligible for final inclusion (Fig. 1). Overall, there were six studies [10, 12–17] comprising Level III and one study that was Level IV evidence. On NOS risk of bias assessment, scores ranged from 3 to 8. Thus, the majority (*n* = 4) of studies were rated as moderate risk for bias, while two studies were ranked as having a low risk for bias and one was determined to have a high risk for bias (Table 1).

### Patient demographics and surgical details

Altogether, data were reported for 774 patients across seven studies, which included six retrospective cohort studies and one retrospective case series. Individual study cohort sizes ranged from 10 to 186 patients, with six of seven studies featuring 60 or more cases and four of seven studies featuring 100 + cases. Cohorts ranged from 22.8 to 60.4% male and mean ages ranged from 42.1 to 56.5 years old. All studies were comparative, and four included comparisons of the RQM to the Kempe incision, five studies focused on evaluating the RQM versus the RA incision type, and one study included both comparisons. Etiologies requiring treatment via DHC included CVA (commonly MCA infarction), SDH (including traumatic aSDH), intraparenchymal hemorrhage, ICH with intraventricular extension, “severe TBI,” traumatic and aneurysmal SAH, and venous sinus thrombosis.

**Fig. 2:**
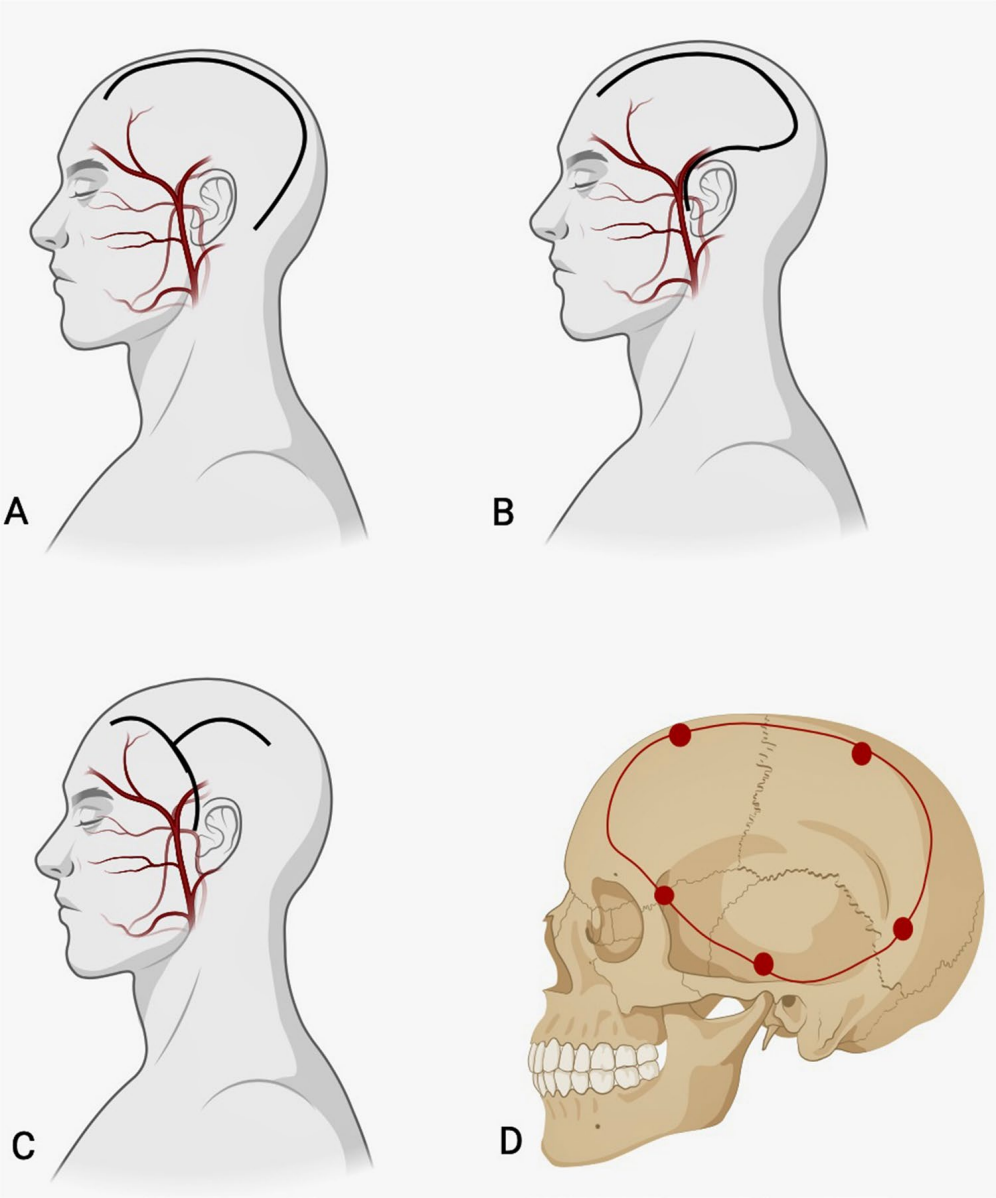
Diagram depicting the relative anatomical positions of the retroauricular (**A**), reverse question mark (**B**), and N-type incisions (**C**) (a true Kempe or T-type incision would traverse along the midline of the calvarium just lateral to the superior sagittal sinus, and an incision perpendicular to this midline scalp incision would be made, forming the T-shape). All incision types enable adequate decompression via the 12 × 15 cm^2^ frontoparietotemporal decompressive hemicraniectomy (**D**), shown here with potential burr hole sites

For studies comparing the RQM and Kempe incisions, the consensus was that both the Kempe and RQM are safe and effective. According to Ordonez-Rubiano [17], the RQM and Kempe are both effective techniques with comparable risk for scalp necrosis, infection, and cosmetic complications. As noted by Abecassis, the Kempe incision may allow the performance of a larger decompression than the RQM [14]. When comparing the RQM and Kempe to the RA, Eltabl and colleagues reported that the RA is a safe substitute for these techniques. Furthermore, they found that the RA can lower rates of post-operative wound complications by preserving blood flow from the STA [16]. Both Dowlati [12] and Veldeman [10] offered similar conclusions in their respective studies, while Dowlati also added that the RA can enable increased calvarial exposure as compared to the classic RQM. As noted by Fruh and colleagues, [13] this effect can secondarily enable performance of an anatomically appropriate DHC that allows for maximal decompression of the temporal base. Finally, Nertengian’s study reported the unique finding that the RA was associated with shorter operative times [15]. In summation, each of the studies comparing the RQM and RA reported positive benefits of the RA, and most reported decreased rates of postoperative wound infection (Fig. 2).

### Meta-analysis: reverse question mark versus alternative (retroauricular and Kempe/T-type scalp incisions) for DHC

**Fig. 3:**
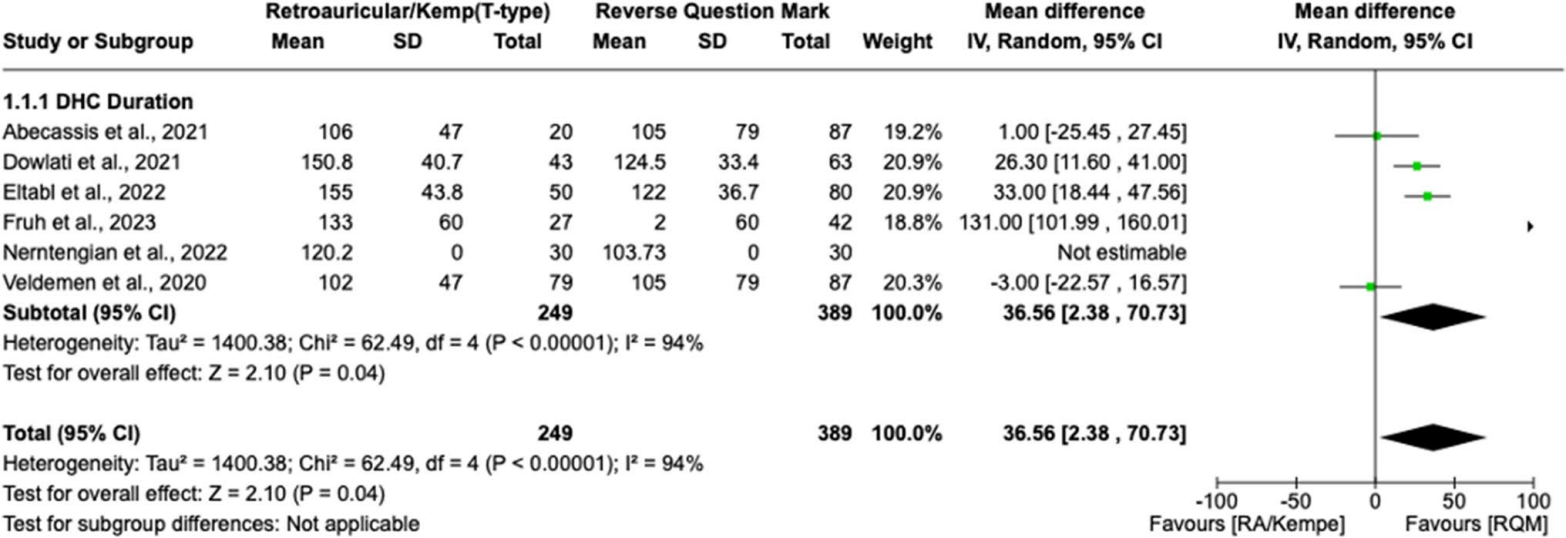
Forest plot demonstrating the statistically significant decrease in operative time when the RQM is used for DHC, as opposed to an alternative incision type (retroauricular and/or Kempe) [MD 36.56, 95% CI 2.38 to 70.73, *I*^2 = 94%, *p* = 0.04]

**Fig. 4:**
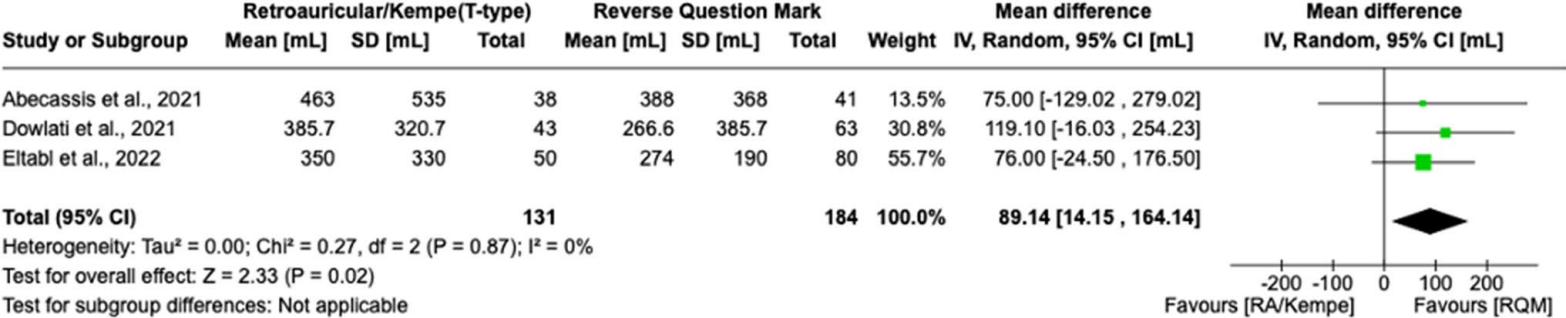
Forest plot demonstrating that mean EBL was significantly lower when the RQM scalp incision was used; specifically, the mean difference in blood loss was 89.14 mL (MD 89.14, 95% CI 14.15 to 164.14, *I*^2 = 0%, *p* = 0.02)

In terms of operative duration, performing scalp incision using the traditional RQM technique shortened surgery by 36.56 min, on average (Fig. 3). This amounted to a statistically significant decrease in operative time when the RQM was used for DHC, as opposed to the alternative incision type (retroauricular and/or Kempe) [MD 36.56, 95% CI 2.38 to 70.73, *I*^2 = 94%, *p* = 0.04]. Additionally, mean EBL (Fig. 4) was significantly lower when the RQM scalp incision was used; specifically, the mean difference in blood loss was 89.14 mL (MD 89.14, 95% CI 14.15 to 164.14, *I*^2 = 0%, *p* = 0.02).

**Fig. 5:**
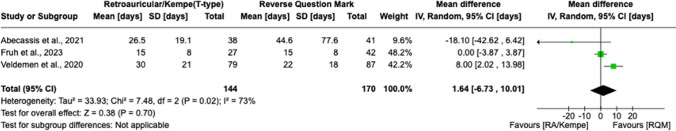
There is no significant association between DHC incision type and mean intensive care unit (ICU) length of stay, as shown by the forest plot below demonstrating a mean difference of 1.64 hospital days (however, this did not reach statistical significance as noted by *p* > 0.05)

**Fig. 6:**
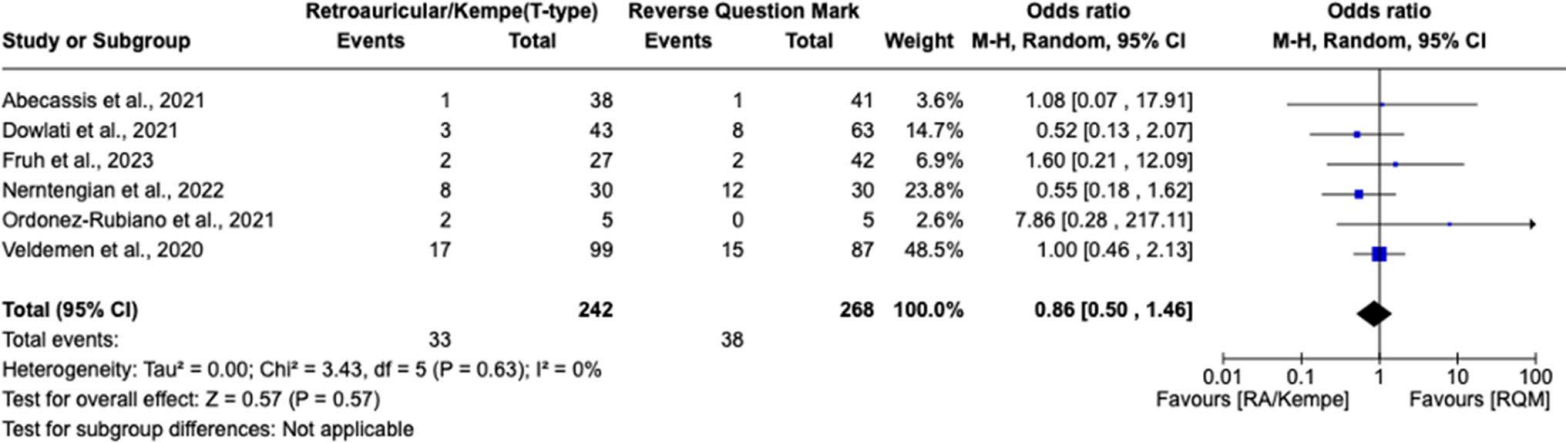
There was no significant difference in predisposition to developing wound complications between the RQM and alternative scalp incision techniques

**Fig. 7:**
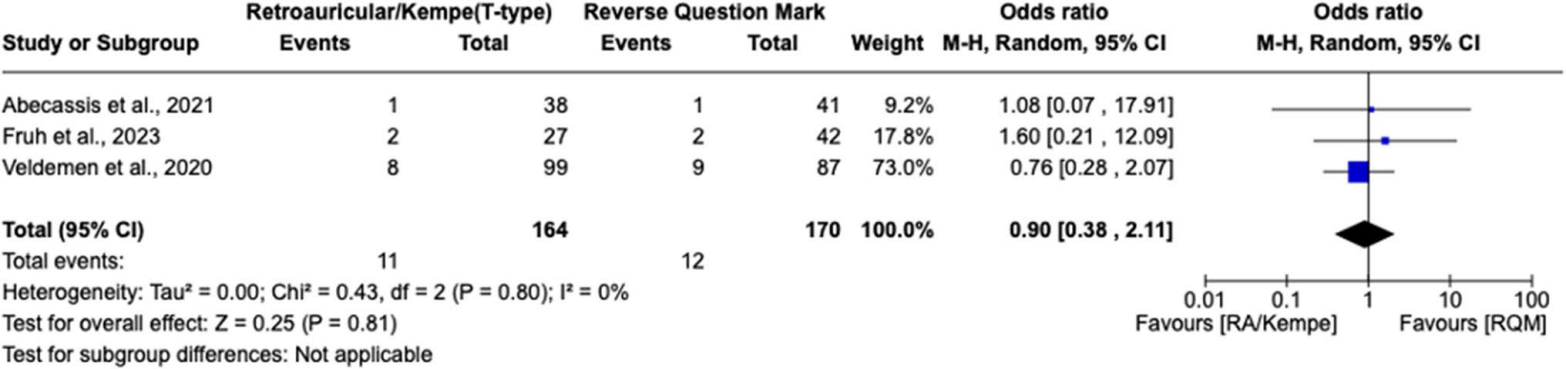
There was no significant difference in predisposition to developing post-operative SSIs between the RQM and alternative scalp incision type cohorts. This is directly contradictory to the results of recent studies, which have reported data supporting the ability of alternative incision techniques (i.e., the retroauricular) to prevent wound infections and other complications

Postoperatively, there was no significant association between DHC incision type and mean intensive care unit (ICU) length of stay (LOS) [MD 1.64 days, 95% CI (− 6.73, 10.01), *I*^2 = 73, *p* = 0.70] (Fig. 5). Nor was there a significant difference in predisposition to developing wound complications (Fig. 6) [OR 0.86, 95% CI 0.50 to 1.46, *I*^2 = 0%, *p* = 0.57] or post-operative SSIs [OR 0.90, 95% CI 0.38 to 2.11, *I*^2 = 0%, *p* = 0.81] between the RQM and retroauricular/Kempe’s incision cohorts (Fig. 7). This is contradictory to the results of individual studies, which have recently touted the ability of alternative incision techniques (i.e., the retroauricular) to prevent wound infections and other complications. Finally, superficial temporal artery (STA) preservation and reoperation rates were collected but could not be analyzed due to insufficient number of studies reporting these outcomes.

## Discussion

Previously, the retroauricular and Kempe incisions have been reported as safe, feasible, and effective methods for performing scalp incision during DHC when compared to the more traditional RQM incision. Retrospective series have suggested that they can provide benefits over the RQM incision while affording a wide enough exposure to enable ample craniectomy for sufficient decompression [7, 18–22]. In the present analysis, we compared outcomes obtained following use of the RQM scalp incision versus the alternative retroauricular and Kempe techniques for emergent DHC procedures with a primary focus on SSIs and wound infections in addition to a secondary focus on EBL, operative times, and mean ICU LOS.

Ultimately, we found that the traditional RQM scalp incision technique is associated with shorter operative times and reduced EBL. To our surprise, utilization of alternative scalp incision techniques such as the retroauricular or T-type (Kempe) did not lead to any reductions in SSIs or wound complications. This result suggests that each of the techniques can be effective for DHC, and that the RQM does not necessarily increase risk for infection as has been previously hypothesized.

Infections and wound complications such as dehiscence are among the most common complications in DHC, even though it is a straightforward procedure that can be performed by junior residents in many cases. One reason that this is the case is that the reverse question mark scalp incision risks damaging the STA—the main blood supply to the large myocutaneous flap that is elevated during this procedure—and additionally places multiple scalp arteries at risk for injury and transection [18, 19]. By comparison, the retroauricular incision minimizes sacrifice of the STA, posterior auricular artery, and branches of the occipital artery. Clearly, if the region is not well perfused because its blood supply is cut off, this may interfere with effective healing [10]. Delays in healing can increase the likelihood of infection and wound dehiscence following the index procedure, hence the motivation for developing alternatives to the RQM [10]. Interestingly, the retroauricular incision has been previously associated with increased hemicraniectomy defect size (in terms of both surface area of skull defect and ratio of skull defect size to skull diameter) as well as a trend toward reduced wound complication rates.

### Background and development: progression of craniectomy as viable intervention

Historically, the use of the “large” decompressive craniectomy for patients with refractory elevated ICP in the setting of TBI was first described by Theodore Kocher around the turn of the twentieth century. As was the case for many neurosurgical innovations, Harvey Cushing subsequently advanced the practice of decompressive craniectomy for immediate reduction of ICP after observing many cases of penetrating TBI during World War I. During the remainder of the twentieth century, the bifrontal craniectomy took hold as the primary DC technique, but was associated with significant morbidity (20% full recovery rate) and mortality (70%) [20, 21]. Thus, it lost popularity in the 1970s and 1980s, even as some groups published good results when using a new technique: the hemicraniectomy [22]. For example, Morantz and colleagues reported their positive experience with DHC in a 1973 study featured in the *Journal of Neurosurgery* in which a cohort of 75 patients underwent DHC for acute epidural and/or subdural hematomas. [22]

Subsequently, during the twenty-first century, bifrontal decompressive craniectomy has been studied in multiple clinical trials, including the RESCUE-ASDH trial. Ultimately, the outcomes obtained from these trials failed to provide definitive guidance for decompressive management of ICP in the setting of TBI, and instead only generated the notion that decompressive craniectomy provides no neuroprotective benefit over medical management for mild-to-moderate intracranial hypertension. However, following the results of the DECRA and RESCUEicp trials, which both provided Level I Evidence, the Congress of Neurological Surgeons and the Brain Trauma Foundations released the 2020 *Guidelines for the Management of Severe Traumatic Brain Injury*, [23] which supports the performance of DHC for patients exhibiting elevated, refractory ICPs in the setting of diffuse parenchymal injury with clinical and radiographic evidence suggestive of impending transtentorial herniation [23]. A summary of the full guidelines is listed in Table 2. Of note, the DHC has also become a routine neurosurgical intervention for malignant cerebral ischemia, aneurysmal subarachnoid hemorrhage, and is under investigation for its efficacy in treating ICH. [14]

**Table 2:** Indications for surgical interventions for TBI by timing and method

*TBI pathology*	*Surgical indications*	*Timing*	*Methods*
*Acute EDH*	EDH > 30 cm^3^ merit emergent evacuation regardless of patient’s GCS score For EDH < 30 cm^3^ with < 15-mm thickness, < 5-mm MLS, and GCS > 8 without focal neurologic deficit, non-operative management indicated. Serial CT scans and frequent neurologic exams	EDH with GCS < 9 with anisocoria merits surgical evacuation as soon as possible	Data are not sufficient to enable clear delineation of one surgical treatment as superior over another
*Acute SDH*	Thickness > 10 mm or MLS > 5 mm on CT merits emergent evacuation regardless of GCS GCS < 9: all merit ICP monitoring GCS < 9 and SDH thickness < 10 mm with MLS < 5 m merit surgical evacuation if: a. GCS score decreases by 2 points from time of injury to time of assessment at hospital b. Patient presents with asymmetric pupils or fixed, dilated pupils c. c. ICP > 20 mmHg	In presence of acute SDH, surgical evacuation should occur as soon as possible if criteria are met	Craniotomy ± bone flap removal and duraplasty
*Traumatic parenchymal lesions*	GCS 6–8, frontal or temporal contusions 20 cm^3^ in volume with MLS ≥ 5 mm or effacement of cisterns on CT merit surgical intervention Any patient with lesion > 50 cm^3^ merits surgical intervention With parenchymal mass lesions, neurological decline referable to the lesion and medically refractory ICP/mass effect on CT—surgical evacuation warranted With parenchymal mass lesions and stable neurologic exam with no evidence of neurological compromise and controlled ICPs, non-operative management with serial exams and CT scans are warranted	Emergent Within 48 h When indications are met	Craniotomy for evacuation of focal mass lesion Bifrontal decompressive craniectomy—option for patients with diffuse, medically refractory posttraumatic cerebral edema driving elevated ICPs Decompressive hemicraniectomy for patients with refractory ICPs and diffuse parenchymal injury with clinical/radiographic evidence of impending transtentorial herniation
*Posterior fossa mass lesions*	Posterior fossa mass on CT Neurological dysfunction/decline referable to lesion, merits surgical intervention Observation with serial imaging appropriate for patients harboring posterior fossa lesions without signs of mass effect or neurological dysfunction Mass effect detected as obliteration of the fourth ventricle, effacement of basal cisterns, and/or presence of obstructive hydrocephalus	Urgent—surgical intervention indicated as soon as possible	Suboccipital craniectomy for evacuation of posterior fossa mass lesions

### The contemporary decompressive hemicraniectomy

When performing the DHC, also referred to as the frontotemporoparietal craniectomy or “Dandy flap,” the patient is positioned supine with the head placed in rigid 3-point fixation or on a horseshoe head holder and rotated 45 to 60° with the option to place an ipsilateral shoulder roll or sandbag [14]. Historically, the bilateral frontal craniectomy was performed to alleviate build-up of pressure, but this has fallen out of favor due to the convenience and ability to preserve half of the skull through the unilateral frontotemporoparietal DHC [14]. This is because the DHC provides better decompression, as it allows the brain to swell laterally to one side, affording the option for quick temporal decompression. Typically, the first step in the exposure when performing the DHC is to incise the scalp in the reverse question mark fashion. The incision begins 1 cm anterior to the tragus and/or posterior root of the zygoma before curving posteriorly, directly above the ear and extending backwards before curving forward near the inion at the midline [23] (Fig. 2). The incision is turned anteriorly along the midline with care to stay lateral to the sagittal sinus and ends at approximately the widow’s peak of the hairline in the frontal scalp region [10]. While scalp is subsequently dissected and retracted, care is taken to preserve the superficial temporal artery (STA) as much as possible through careful dissection, though this is not always possible during emergent surgery and injury to the STA is a source of poor wound healing outcomes.

In a subtle variation of the reverse question-mark incision, the “retroauricular” incision begins “behind the ear” as its name suggests (posterior to the base of the mastoid, specifically) and then courses posteriorly before curving anteriorly at the lambdoidal suture and then continuing anteriorly to the widow’s peak of the hairline, again staying lateral to the superior sagittal sinus [24]. Termed an “altered posterior question-mark” incision by Veldeman and colleagues, it is intended to help prevent injury to the STA and to facilitate decompression of the temporal base.

As previously discussed, the retroauricular scalp incision appears to provide superior overall and temporal lobe decompression while demonstrating a trend toward reduced rates of wound complications [12]. In a prior study, skull surface defect areas associated with the retroauricular incision were, on average, 9.2 cm^2^ larger than defect areas associated with RQM scalp incisions (*p* < 0.001) [12]. Furthermore, because the retroauricular incision is more likely to preserve the blood supply to the scalp (STA), it is theoretically more conducive to wound healing. This is particularly important in DHC because adequate perfusion must be maintained following the index procedure as well as through the completion of the cranioplasty [25]. Importantly, the retroauricular incision technique does not interfere with the ultimate extent of decompression obtained through the DHC. Moreover, it enables comparable temporal lobe decompression as demonstrated by lack of signs of herniation on postoperative CT scans in prior studies. [13]

In addition to providing comparable decompression, the retroauricular incision was found to reduce rates of postoperative infection and cranioplasty failure [10]. These findings can be attributed to the skin flap preserving properties of the retroauricular incision, which does not interfere with the perfusion supplied by the STA (and also partially preserves the occipital artery, another contributor to perfusion of the vascularized skin flap).

Insofar as the Kempe incision—also termed the “T-type” or “T-bar” incision—is concerned, special attention should be paid when securing the cranium with Mayfield pins as it may be habitual (for those used to pinning the cranium for the RQM) to incorrectly place a posterior pin where it would normally lie when an RQM is being performed [26]. The problem with such a misplacement is that this misplaced posterior pin would interfere with the midline incision during performance of the Kempe incision. However, special attention must also be paid to ensure that—while avoiding midline with the pin—adequate purchase is still obtained so that the patient’s head does not slip out of fixation [27]. Beginning posteriorly at the inion/external occipital protuberance, the scalp incision follows the course of the sagittal sinus to the front of the hairline. This incision is supplemented by perpendicular incision that generates the T-bar. This incision begins 1–2 cm anterior to the tragus at the temporal root of the zygoma, near the region of the starting point for the RQM technique. Care must be taken not to violate the STA just as is the case for the RQM incision [14]. In their 2021 series featuring 79 DC patients from 2015 to 2020, Abecassis and colleagues [25] share that the main issues with the RQM—though it does provide adequate exposure for decompression when craniectomy is performed in the 12 × 15 cm^2^ manner—include that it involves sacrificing the occipital and posterior auricular arteries which leads to higher risk for wound infections and other complications.

When compared to the traditional RQM, the Kempe incision is associated with comparable outcomes (wound infection rates, estimated blood loss, surgical duration, wound dehiscence, cranioplasty success rates) for treatment of TBI and stroke [14]. Additionally, it has demonstrated that it enables a larger decompression than is made possible by the RQM. Furthermore, it comes with the added advantage of flexibility: the Kempe incision can easily be converted to an incision suitable for performance of bilateral or bifrontal craniectomy, an important alternative option for decompression in emergent scenarios [14]. Additionally, the motivation behind the implementation of the Ludwig Kempe hemispherectomy incision came from military neurosurgeons. They have suggested that the T-shaped incision enables maximal decompression when the craniotomy is performed and that the incision preserves vascular pedicles that supply the scalp (which theoretically should help minimize complications such as wound infection and/or dehiscence). According to Abecassis and colleagues, the Kempe incision is particularly useful when a bilateral pathology is suspected or complex facial fractures are noted on exam that may require the performance of a bifrontal craniotomy for orbital reconstruction (again, the Kempe preserves the vascular supply to the scalp in the involved regions) [14]. Finally, when the intracranial pathology is primarily localized to the posterior parietal or occipital regions, the Kempe incision enables a more targeted posterior decompression. [14]

There is a particularly high risk for wound dehiscence to occur in a gravity-dependent manner along the posterior margin of the traditional RQM incision [28]. Interestingly, these claims have been asserted and reported across individual studies, but when pooling the results of studies comparing SSI and wound complications by DHC scalp incision type via meta-analysis, we found these rates to be equivocal. This finding is based upon an analysis that includes scalp incision technique as the lone independent variable assessed. Therefore, the findings reported here must be interpreted in light of the fact that other factors—including but not limited to surgical technique, wound closure technique, quality of post-decompressive management to avoid cerebral edema and promote normal healing of the scalp wound, and the role that differences in prophylactic antibiotic use play in development of SSIs—may have had an unknown and unquantifiable confounding effect. Ultimately, this indicates that further studies are needed, ideally prospective in nature and featuring larger cohorts, so that a more valid and statistically powered evidentiary base can be established to confirm the findings we report.

### Limitations

We acknowledge several limitations to the present study. First, though the studies featured in the present analysis did not report DC flap size in a consistent manner, this is a related and highly relevant factor that has been debated extensively. Although the neurosurgical community has accepted the 12 × 15 cm^2^ hemicraniectomy size as a procedural consensus, this topic is still widely debated. In fact, it is logical to question whether DHC size variation could have played a role in the heterogeneity of SSI and wound complication results reported across studies. Unfortunately, there is no definitive way to determine whether flap size may have been a confounding factor or effect modifier.

Moreover, it is likely that the most important effect on ultimate surgical outcome would have been the relationship between incision type and DHC bone flap size. As the quality and orientation of the surgical incision directly correlate with the maximum DHC size that can be performed, it would be important to understand the effect of incision type on the range of the craniectomy size that can be performed. Furthermore, it would be important to know whether any of the incision types impose limitations upon temporal lobe decompression, including the ability to remove additional temporal bone if needed intraoperatively. Many surgeons would likely opt for the incision type that provides maximum calvarium exposure and thereby enables optimal removal of the hemicranium. This is paramount to achieving adequate decompression and may help ensure that the surgeon can successfully decompress the temporal lobe (arguably the most important component of the procedure). [12]

In the fourth edition of their severe TBI guidelines, the Brain Trauma Foundation recommended that DHCs be no smaller than 12 × 15 cm^2^ as a level 2A recommendation intended to optimize decompression and minimize the risk for severe herniation at the edge of the bone flap. Interestingly, the quantity of medical literature investigating the role of bone flap size in DHC is underwhelming. Although the 12 × 15 cm^2^ DHC is widely regarded as standard of care, this is based on the assertion that a more limited craniectomy is suboptimal. The 12 × 15 cm^2^ DHC is therefore not based upon precise, clearly delineated and quantifiable metrics. There is still no certainty as to whether bone flap surface area is truly an independent predictor of clinical outcomes [29]. Because this is the case, this adds an extra layer of difficulty to identifying the optimal scalp incision technique.

One of the challenges in determining appropriate craniectomy flap size is that the 12 cm × 15 cm^2^ measurement guideline does not account for variations in patient head size. In 2020, Schur and colleagues released the results of an inquisitive study in which they examined the optimal bone flap size for DHC in a patient-specific manner by tailoring the flap size to individual patient cranial vault size [30]. Their methodology for tracking the effect of bone flap size relative to head size was to track the ratio of flap circumference to each patient’s skull hemi-circumference and then to analyze the correlation between this ratio and relevant clinical variables (hospital and ICU LOS, need for hypertonic infusion, ICP control, and more). Interestingly, ICPs were found to be significantly lower in patients whose bone flap circumference:skull hemicircumference ratio was > 65% (*p* = 0.01) [30]. In other words, the results of the study suggested that a larger craniectomy flap that is approximately two-thirds of the hemicircumference of the patient’s skull enables improved ICP control—with reduced need for administration of mannitol—postoperatively. Although these results are potentially meaningful and may warrant further investigation, they could not be extrapolated beyond ICP control; in other words, improved ICP control was not correlated with differences in clinical outcomes [30]. In summation, both scalp incision type and craniectomy bone flap size merit further investigation to determine optimal DHC parameters. Certainly, the idea of patient-specific DHCs is appealing and could lead to improvements in neurocritical care of patients with TBI and/or refractory ICPs.

Additionally, the present meta-analysis was limited in scope to just seven qualifying studies, leaving significant room for publication bias to influence the results of this study. Furthermore, this means that even in the absence of publication bias, the analysis is not optimally powered, and this should be considered when interpreting the results reported herein. Furthermore, it is difficult to determine the way in which heterogeneity across studies—in terms of clinical and operative factors such as surgical technique and/or institutional protocols—could have influenced findings. For example, as previously mentioned, DHC bone flap size can have a significant effect on the success of the procedure and thus serve as a potential source of confound or effect modification. Nonetheless, our study represents the first focused systematic review and meta-analysis on the potential association between different DHC scalp incision techniques and rates of SSI/wound complications, among other postoperative outcomes.

## Conclusion

Ultimately, it is a positive that there have been numerous attempts to refine techniques for decompression of herniating brain in situations that can mean the difference between life and death. Previous literature has suggested that SSIs, wound dehiscence, and other postoperative complications can be minimized when alternative scalp incision techniques—such as the retroauricular or Kempe incisions—are used instead of the classic reverse question mark incision. However, on meta-analysis, there does not appear to be a significant difference between scalp incision techniques insofar as these outcomes are concerned (of note, however, our analysis for SSI was likely affected by statistical underpowering). At present, it appears that, at the very least, outcomes following DHC can be improved by ensuring that the bone flap is large enough to enable sufficient cerebral expansion, and in particular decompression of the temporal fossa, while ensuring that the craniectomy is performed without breaching dural venous sinus. Regardless of the specific scalp incision technique used, surgeons can control many other variables—including but not limited to achieving adequate temporal decompression, the use of prophylactic antibiotics, and wound closure techniques—that can each decrease the likelihood for occurrence of SSI or wound complications.

## Data Availability

Datasets are not publically available and can be obtained upon further request.

## Declarations

### Preparation

This protocol was not registered, nor was it reported in a publicly accessible repository prior to submission.
